# Physician Men Leaders in Emergency Medicine Bearing Witness to Gender-Based Discrimination

**DOI:** 10.1001/jamanetworkopen.2022.49555

**Published:** 2023-01-05

**Authors:** Maya S. Iyer, Kalah Wilson, Claire Draucker, Cherri Hobgood

**Affiliations:** 1 Division of Emergency Medicine, Department of Pediatrics, Nationwide Children’s Hospital, The Ohio State University College of Medicine, Columbus, Ohio; 2 Department of Sociology North Carolina State University, Raleigh, North Carolina; 3 Division of Community and Health Systems Indiana University School of Nursing, Indianapolis; 4 Department of Emergency Medicine, University of North Carolina School of Medicine, Chapel Hill

## Abstract

**Importance:**

Approximately 60% of women physicians in emergency medicine (EM) experience gender-based discrimination (GBD). Women physicians are also more likely to experience GBD than men physicians, particularly from patients, other physicians, or nursing staff.

**Objective:**

To describe the responses of men who are academic department chairs in EM to GBD directed toward a woman colleague.

**Design, Setting, and Participants:**

This qualitative study was a secondary data analysis drawn from interviews of men EM academic department chairs at 18 sites who participated in a qualitative descriptive study between April 2020 and February 2021 on their perceptions of the influence of gender and leadership in academic medicine. Narrative data related to GBD were extracted and coded using conventional content analysis. Codes were clustered into themes and subthemes and summarized. Data were analyzed from November to December 2021.

**Exposure:**

Semistructured interviews conducted via teleconferencing.

**Main Outcomes and Measures:**

Qualitative findings identifying experiences witnessing or learning about incidents of GBD against women colleagues, the impact of these observations, and personal or leadership actions taken in response to their observations.

**Results:**

All 18 men participants (mean [SD] age, 52.2 [7.5] years; mean [SD] time as a department chair, 7.2 [5.1] years) discussed witnessing or learning about incidents of GBD against women colleagues. The participant narratives revealed 3 themes: emotional responses to GBD, actions they took to address GBD, and reasons for not taking action to address GBD. When witnessing GBD, participants felt anger, disbelief, guilt, and shame. To take action, they served as upstanders, confronted and reported discrimination, provided faculty development on GBD, or enforced “zero-tolerance” policies. At times they did not take action because they did not believe the GBD warranted a response, perceived a power differential or an unsupportive institutional culture, or sought self-preservation.

**Conclusions and Relevance:**

In this qualitative study of men physician leaders, we found all participants reported feeling troubled by GBD against women colleagues and, if possible, took action to address the discrimination. At times they did not take action because of unsupportive workplace cultures. These findings suggest that institutional culture change that supports the interventions of upstanders and does not tolerate GBD is needed.

## Key Points

**Question:**

How do men physician leaders perceive and respond to gender-based discrimination (GBD) against women colleagues?

**Findings:**

In this qualitative study including 18 men academic department chairs in emergency medicine, all study participants reported either witnessing or learning of incidents of GBD against women colleagues. Participants reported that these experiences with GBD stimulated powerful emotional responses and presented a choice about taking action.

**Meaning:**

These findings suggest that GBD against women impacts targets and observers and institutional cultures must be intolerant of GBD to support all physicians.

## Teaser

This qualitative study describes the experiences and responses of men academic department chairs in emergency medicine regarding gender-based discrimination directed toward a woman colleague.

## Introduction

Gender-based discrimination (GBD) against women physicians in all academic medical specialties, including emergency medicine (EM), is well documented.^1,2^ GBD is defined as harmful actions or comments against individuals based on gender and includes bias, overt mistreatment, inequitable treatment, and sexual harassment.^1,3^ Women report experiencing GBD, such as sexual harassment, patronizing actions, comments on appearance, sexual advances, and innuendos.^4^ In EM, women report high rates of GBD: 52.6% of women in EM report being sexually harassed, 25% report gender-based disrespect, and 9.5% report at least 1 encounter of sexual assault by a colleague or supervisor during their career.^1,5,6,7^ Women physicians are more likely to experience GBD than men, particularly from patients, other physicians, or nursing staff.^1,8^

Despite GBD’s prevalence, women physicians report only approximately 5% of incidents, often due to the belief that nothing will be done or changed or the fear of retaliation.^8,9,10^ Research suggests that cultures that promote inclusivity and support upstanders (ie, persons who intervene on behalf of others) are needed to end GBD.^11^ Multiple studies describe the experiences of women physicians who experience GBD^8,12,13,14^; however, little is known about the experiences of men physicians who witness, learn of, or perpetrate GBD toward women. The objective of this study is to describe the self-reported responses of men who are academic department chairs in EM to GBD directed toward a woman colleague.

## Methods

This secondary qualitative study was conducted on data drawn from a larger study (primary study).^15,16^ This study was approved by the institutional review boards of Indiana University and the University of North Carolina Schools of Medicine. All participants provided verbal consent. This study followed the Consolidated Criteria for Reporting Qualitative Research (COREQ) reporting guideline.

### Primary Study

The primary study was a qualitative descriptive study of gender and leadership in academic medicine.^15,16^ The qualitative descriptive approach used purposive sampling, semistructured interviews, and content analysis to obtain a descriptive summary of the phenomenon of interest.

#### Research Team

The primary study research team comprised a former EM department chair, a qualitative methodology expert, and several public health researchers. To mitigate bias, all team members completed a personal positionality memo reflecting on their relationship to the discipline, data, and participants, thus addressing reflexivity of the research team and COREQ guidelines. These memos guided exploration of team member biases that might affect result credibility.

#### Selection of Participants

The primary study^15,16^ used purposive criterion-based sampling to identify 19 women and 18 men EM academic department chairs. Preliminary lists of chairs were compiled from national organizations,^6^ and university websites verified eligibility. Potential participants were emailed study descriptions and invitations. Nonresponders received 2 email reminders. Twenty women chairs were identified, and 19 accepted the study invitation. Seventy-seven men chairs were identified, 37 were randomly selected using Research Randomizer,^17^ and 18 agreed to participate. Participants were informed of the study’s purpose and that confidential interviews would be conducted and recorded with Zoom videoconferencing software (Zoom Video Communications).

#### Data Collection

The research team developed, piloted, and iteratively revised an 11-item structured interview guide. The senior author (C.H.), a former EM chair, known to all study participants because of her leadership status, conducted individual 1- to 2-hour interviews between April 2020 and February 2021. Participant responses guided unrestricted discussion. Recordings were deidentified, transcribed, and anonymized. Participants provided demographic information, including age, gender identity, years in position, and institution.

#### Analysis

Numeric demographic data were summarized using means and medians and categorical data using frequencies and percentages. Using NVivo software version 12 (QSR International), qualitative data were inductively coded.^18^ Two team members independently coded a 9-transcript subsample. Recurring codes were identified using a process of comparison and consensus to iteratively develop an initial coding framework. Team members used this framework to independently code all remaining transcripts. If new codes emerged or required clarification, revision occurred via consensus. To ensure coding accuracy, 15% of transcripts were randomly selected, double-coded, and compared using the κ coefficient. Any κ scores less than 0.6 were reviewed and resolved by consensus. C.H. summarized the findings, and the team confirmed their accuracy. Participant checks were performed by sharing themes and representative quotes with participants for feedback and verification. Previously published methods and results from this analysis include a description of leadership challenges experienced by women academic department chairs in EM^19^ and gender differences in leadership emergence.^15^ The research team noted that all the men, and rarely any of the women, participants discussed their experiences with observing or learning of GBD, often in a powerful manner, identifying an opportunity to conduct a secondary analysis exploring how an advantaged group (men leaders) respond to GBD toward a less advantaged group (women colleagues).

### Secondary Analysis

#### Research Team

The research team for the secondary analysis included the primary study authors, a pediatric EM physician, and a sociology graduate student. New members completed a personal positionality memo, and team debriefings explored any biases that might affect result credibility.

#### Participants and Data Collection

The secondary analysis used the transcripts of the 18 men participants. We used 4 interview questions (No. 3, 7, 8, 9) (eAppendix in Supplement 1) that explored the work environment, observed or disclosed incidents of sexual harassment, microinequities, and microaggressions produced most of the data; however, other references to GBD interspersed throughout the interviews were included in the data set.

### Data Analysis

Team members (M.S.I., K.W., C.H.) independently reexamined participant transcripts and extracted GBD-related remarks. These included participant responses to incidents of observed, heard about, or perpetrated GBD against woman colleagues. Team comparison of extracted remarks established consensus for data set inclusion.

Extracted remarks were independently inductively coded with labels reflecting their meaning.^18^ Team discussion and consensus clustered labeled phrases and developed themes. Because the coding was not highly interpretative and focused on the participants’ surface meaning, coding disagreement or ambiguity was easily resolved. The lead author (M.S.I.) prepared narrative theme descriptions, and team members confirmed their accuracy. Data were analyzed from November to December 2021.

## Results

All 18 participants acknowledged witnessing or learning about GBD targeted toward a woman colleague. All participants were cisgender men, mean (SD) age was 52.2 (7.5) years, and the mean (SD) tenure as department chair was 7.2 (5.1) years. No participants discussed knowingly perpetrating GBD. Incidents they described included sexist and derogatory comments, sexist humor, inappropriate sexual interactions, belittling of ideas or contributions, and discrimination in employment practices (eg, promotions, appointments). One participant said, “The most obvious manifestations of gender disparities [in academic medicine] is in the way people are treated and interacted with every day, you know, on the floor or in committee meetings” (participant 8). The analysis revealed 3 main themes that reflected the participants’ responses to the GBD to which they bore witness: (1) the emotions they experienced, (2) the actions they took, and (3) their reasons for not taking action.

### Emotions in Response to GBD

Participants discussed 4 emotions related to witnessing GBD. They reported experiencing anger at the entitlement of their colleagues who engaged in discrimination, disbelief that it was occurring, guilt that they did not use their power to stop it, and shame that they had participated in such incidents in the past. In some instances, the emotions were quite powerful. Participant 4 stated “Am I going to be able to sleep tonight and be able to tell my son that I’m a good person? Because it really gets at your core values when you’re a witness to these things.” Table 1 provides supportive quotes.

**Table 1.** Emotions Reported in Response to Gender-Based Discrimination

Emotion	Participant No.^a^	Illustrative quote
Anger	18	“I have learned that the way women are treated in the world is completely different than the way I am treated in the world. I mean, it’s jaw-dropping. It’s what’s said, what’s not said, what’s done, and what’s not done. It’s active, passive, universal. It’s ridiculous the extent of gender disparity in academic medicine.”
Disbelief	9	“I was with a female colleague, and a male colleague who knows us both [publicly committed gender-based discrimination]. I’ll be honest with you, I was quiet, because I was just shocked and I was so embarrassed for [my female colleague] that I didn’t say a word, and she just looked down. I can’t imagine how many times that has happened.”
Guilt	17	“When he said it [sexist comment] there was only one female in the room, and I looked straight at her, and we met eyes and then our hearts just sank. We both just looked at the ground, and I feel guilty about being complicit and not speaking up at that moment.”
Shame	8	“I’m ashamed of it, and I probably participated in it. Especially, when I started in emergency medicine, sexual jokes were very, very frequent, and I did see what I would call sexual harassment and inappropriate comments that were made, and I was not very vocal.”

^a^ For anonymity, participants are identified with numbers.

### Actions Taken to Address GBD

Participants discussed 4 types of actions they took to address GBD. Actions were most likely if participants felt they had status or institutional support to have their voices heard. In some instances, participants served as an upstander by speaking up for women targets of GBD. For example, some participants reported that they “called out” others who made derogatory or harassing comments toward women colleagues. In some cases, participants said they praised women who were being maligned. One participant pushed back against colleagues who were putting down a woman leader and said, “Well, I like her substrate. I think she is wildly smart and very deliberate” (participant 9). In cases participants considered especially egregious, such as ongoing mistreatment and sexual harassment, they reported confronting the offenders and reporting their actions to designated officers in the institution. Other participants reported that they enforced a “zero-tolerance” policy against discrimination in their departments. This enforcement included disciplining or dismissing offenders, with the aim of creating a culture of respect for women. One participant stated, “Our job is to recognize, clearly identify, and not circumvent the corrective response” (participant 5). Finally, some participants said they worked on changing the culture of medicine by increasing the representation of women through faculty recruitment, sponsoring and mentoring women and minoritized faculty, and providing leadership coaching and workshops designed to support gender equity and decrease GBD. Illustrative quotes are provided in Table 2.

**Table 2.** Actions Taken in Response to Gender-Based Discrimination

Action used	Participant No.^a^	Illustrative quote
Served as an upstander	1	“I have had comments made to me about females in the workplace that would be viewed as harassment, and the last 2 times that happened to me, I just called it right there, said it for what it was.”
	15	“I just said, ‘Hey, that’s just not appropriate for the workplace or really appropriate for anyplace.’ And it was surprisingly well-taken.”
Confronted and reported discrimination	9	“I was torn up about what to do. But this [observed mistreatment] was not something I could be a party to. So, I went and I talked to him about it, and I said, ‘It was not okay, and it can’t stay between us.’ So, I got someone from the Office of Professionalism,… and it turns out he did this kind of thing all the time.”
	17	“A resident physician came to me and said, ‘Dr. X, when he talks to me, he looks at my breasts.’ I reported that.”
Changing the culture of medicine	2	“The dean asked me how I built a very large department to be 50/50 [gender mix] I said, ‘You just hire the most qualified people for the job,’ but I am conscious of the number of male and females I’m hiring.”
	9	“I hired a coach for one of my women faculty members, and it was transformative, she went to the next level. And now, she’s an institutional leader, and I feel comfortable being her sponsor, too, like saying, ‘Hey, we’ve got this woman who’s really smart. You should take a look at her for A, B, and C.’”
	3	“For one [woman faculty member], the thing that helped a lot was to get a coach for her. It was an objective third party to say, ‘What is it that you’re doing right?’ For another one [woman faculty member], I set up some mentoring, and now she feels innovative and creative and that’s helped.”
	10	“We’ve started having diversity and inclusion sessions monthly, where everybody in the department gets together and we have a topic that we’ll go through, then we have open discussions about any issues. I think it’s been a very healthy thing within the department.”
	8	“One of the things that has worked for women’s development is seminars or groups that I support to raise women up and help to deal with some of the very nitty-gritty kinds of issues that are just more prevalent for women than for men.”
Enforced a zero-tolerance policy	12	“I’ve made it clear to my faculty I have a zero-tolerance for [sexual harassment]. So we’ve had allegations in the last 2 or 3 years, and those faculty members are no longer here.”
	3	“I think we’ve tried to create a culture where if anything happens, that it’s easily reported, and it doesn’t escalate, and it’s not tolerated.”

^a^ For anonymity, participants are identified with numbers.

### Reasons for Not Acting Against GBD

Conversely, the participants discussed 4 reasons why they did not act when they witnessed GBD. Sometimes, the participants did not act because they did not think the incident warranted a response. They did not judge the incident was severe, believed the woman took could take care of it herself, or the incident passed before they could respond. One participant said “I’m not that integrated, I’m not that put together that I can always be completely in the moment and have the right response” (participant 8). When participants did not act more often, they perceived a power differential in which the person engaging in the discrimination had more authority. Despite wanting to “do the right thing,” participants believed any action they took would be fruitless. Others reported that they did not take action because they perceived an unsupportive institutional culture. They experienced their institutions as inherently biased, failing to take definitive action even against persons who did “horrendous” things. Some participants said they failed to take action because of the need for self-preservation. They feared and did not want to experience negative consequences to their institutional standing because they acted against GBD. As a result, they took a conservative approach to GBD and chose to “fall in line” with those with more power. Illustrative quotes are provided in Table 3.

**Table 3.** Reasons Reported for Not Acting Against Gender-Based Discrimination

Reason for inaction	Participant No.^a^	Illustrative quote
Did not feel incident warranted a response	10	“When she [woman chair] was interrupted or another chair tried to step in and run the meeting, she would just continue running the meeting. She didn’t need anyone to step in and protect her in any way. She managed it.”
	11	“Sometimes people just say subtle things, not necessarily directed at anyone. I don’t think it’s intentional and oftentimes people are not even really paying attention, so they are unrecognized.”
Perceived a power differential	17	“There’s a utter lack of strong or of female leadership within our hospital. When I discuss this with our with our CEO, he says, ‘You know sometimes I’m powerless. I have to report to the board of trustees.’”
	4	“If it’s somebody who’s got a lot of power over me, how do I engage in that situation? I hope I do the right thing as much as I can. But sometimes the power differential’s just too large.”
Perceived an unsupportive institutional culture	6	“It’s not just about running out and finding more people of color to work in our department and hiring more women, it’s about fixing the situation so that it’s a place where people of color and women can work safely. I thought I’d be able to just fix it, ‘Oh, it’ll make our group more diverse if we just hire people,’ but our system is not really built for that yet.”
	4	“The majority of the time that I’ve seen this in the past 10 years is on the professionalism committee, nobody gets fired, everybody goes to training. Horrendous things—you can’t even repeat them, they’re so vulgar. You know, these kinds of things are happening, and it’s amazing to me what little happens.”
Self-preservation	1	“When things [incidents of gender mistreatment] happen, a lot of times, the blind eye is turned, and there are few consequences. You have to play that, very carefully and I ensure my approach is fairly conservative. I am always careful. I’m not gonna jump up and down and cry, ‘This is unfair!’”
	7	“The biases that are out there. And it’s awful… but at the end of the day, when it’s your leader, it is what it is. You gotta fall in line, you know.”

^a^ For anonymity, participants are identified with numbers.

## Discussion

The results of this qualitative study show that all participants reported having witnessed GBD against women colleagues and that this experience generally troubled them. While some took action in response to the discrimination, many did not, despite being men, department chairs, and in authority positions. Resonate with prior scholarship, most participants attributed their inaction to the culture of academic medicine. As a field, academic medicine often operates on principles of hierarchy, a set of integrated levels within which team members are ranked by their disciplines and levels of authority.^1,20^ These types of hierarchies often overvalue the contributions of some individuals and dismiss the contributions of others, create competition, operate on authority gradients that legitimize mistreatment, create a negative working environment, and lead to moral distress.^1,21^ In these cultures, persons, irrespective of gender, likely find voicing GBD-related concerns difficult due to fear of loss of employment or position, retaliation, or social exclusion. This even occurs for men leaders, who, like our high-ranking participants, may find that “silence is pervasive in organizations due to the widely shared belief that speaking up about sensitive issues is futile or even dangerous.”^22,23^

While the perceptions of GBD among women who are targeted are well-documented,^1,3,7^ our findings add to the literature by addressing the perceptions of GBD among an advantaged group, men leaders, who bear witness to it. Witnessing GBD produced negative emotional responses, including feelings of shame, guilt, disbelief, and anger, in these men participants. These types of emotional responses are common responses to trauma and often occur when individuals fail to meet their own expectations, engage in negative internal dialogue, or experience a sense of uncertainty about their fitness for the role they occupy.^24,25^ In other facets of academic medicine, similar emotional responses have been found to be associated with destabilizing effects among those who experience them. These effects include a sense of humiliation, job ineffectiveness, loss of productivity, lack of empathy, breaches in professionalism, and, ultimately, disengagement or disinterest in the profession.^26,27^ While the participants in our study did not identify these outcomes, the literature suggests that individuals who are not men and are not holding leadership positions experience even greater stress from witnessing GBD, ultimately associated with burnout and decreased quality of patient care.^1,28,29^ Therefore, our results suggest that all witnesses to GBD in the academic medicine environment may endure severe workplace stress. Identifying and discussing the pervasive influence of GBD is critical to addressing the dysfunctional power hierarchies limiting culture change across all aspects of academic medicine.

The findings of the study suggest several approaches to combat GBD, such as facilitating the advancement of women in medicine. Cultivating women to become leaders is critical to institutional growth and success through generating increased organizational productivity, improving healthy policy, national prosperity and quality of life, and increasing patient satisfaction.^30,31,32^ Participants suggested and enacted initiatives to hire, retain, and advance women leaders to address gender-based dysfunctional hierarchies that allow GBD to flourish. Increasing women leaders’ representation in academic medicine enhances equity, value, and respect for women.^33,34,35^

Implementing comprehensive strategies to promote an inclusive environment is also necessary to disrupt GBD. The National Institutes for Health (NIH) Strategy for Inclusive Excellence reports that advancement of women and individuals from other underrepresented groups requires accountability and transparency; clear metrics of inclusion, diversity, and equity; tracking and evaluation of such metrics.^33^ To enhance the utility of data to root out disparities and address them directly, the NIH disaggregates data by department and expands beyond basic demographics to other metrics, including salary, space, personnel and additional support, speaking invitations, and work-life integration policies. A senior NIH faculty committee oversees and transparently reports on the process.^33^

Similarly, the Association of American Medical Colleges (AAMC) has identified common strategies that contribute to creating a safe and inclusive environment. These include a “zero-tolerance culture” supported by improved reporting, transparency, leadership accountability, and sanctions for offenders.^1^ Study participants endorsed zero-tolerance policies and systemic changes to the culture of medicine that promote individual and organizational accountability for eliminating GBD.^1^ Our findings also indicate the need for more robust institutional safeguards for persons who wish to serve as allies but fear retaliation or social exclusion.

In addition, our findings indicate a need for institutions to provide comprehensive skill development for GBD recognition, even in its less overt forms, and training in effective upstander and allyship behavior.^36,37^ Existing exemplar programs have been instituted at the NIH for upstander and implicit bias training and at Vanderbilt University to identify, measure, and address unprofessional behaviors.^33,38^ These organizational strategies normalize inclusivity and use transparent processes to address unprofessional behaviors, thereby promoting a culture of civility.

Finally, allyship is strategy to decrease GBD. Allies are “members of an advantaged group committed to building relationships with women, expressing as little sexism in their own behavior as possible, understanding [their own] social privilege, and demonstrating active efforts to address gender inequities.”^36^ Allies support institutional policies that ensure women’s inclusion on strategic committees and in leadership, role model respectful interactions, and mentor and sponsor women faculty.^34,36^Above all, the literature shows that allyship is supported when allies of any gender are viewed as “critical actor” leaders, who individually and collectively have the commitment and power to create gender-equitable cultures.^39^

### Limitations

This study has some limitations. Because this study is a secondary analysis from a primary study of gender and leadership in academic medicine, our analysis did not permit targeted probing on GBD. For example, because GBD was not the interview focus, participants may have had additional experiences with GBD that they did not discuss and that were not elicited and explored in-depth. In particular, the most salient experience with GBD among women leaders in the primary study was experiencing GBD themselves.^15^ As such, we could not probe if women primary study participants did, in fact, witness GBD and identify their respective responses. The men participants openly discussed their experiences bearing witness to GBD, providing an opportunity to explore how an advantaged group (men leaders) responds to GBD toward a less advantaged group (women colleagues).

The sample also created some study limitations. Given that all the participants were academic department chairs in EM, their responses may not represent chairs in other specialties. However, the in-depth descriptions and abundant examples of GBD spontaneously provided by all participants suggest that witnessing these events is commonplace. In addition, men in junior ranks were not interviewed. This group may be more like to intervene when witnessing GBD due to generational differences, or, conversely, less likely to intervene because they have less power in the institution than their senior colleagues and thus, are more vulnerable to retaliation. Individuals who are transgender and nonbinary also have experiences with GBD^2^; however, these perspectives and experiences were not represented in our sample.^40^

The gender and experience of the research team may have introduced bias. A priori positional statements, task segregation, the inclusion of nonmedical research team members, theme verification, and member checking served to counterbalance these biases. Using a single interviewer, a woman former EM chair, may also be a limitation; however, it may also have provided commonality between participant and interviewer, sanctioning participants to divulge information that is ostensibly not socially desirable for men leaders, such as inaction in the face of GBD and strong emotional responses. Additionally, none of the participants discussed engaging in GBD. The narratives of physicians who engage in these behaviors would add richness to our understanding of the phenomenon.

## Conclusions

In this qualitative study, the men we interviewed all reported having witnessed GBD, and most reported experiencing negative emotional responses as a result. While many participants wanted to do the right thing and serve as allies to women by taking actions to combat GBD, they often felt they were stymied by a culture in academic medicine in which gender equity was not valued and GBD was tolerated. Medical organizations and institutions must change the collective culture and mindset to value the contributions of both men and women physicians. Only then will all physicians work in emotionally safe environments and be empowered to speak against and end GBD.

## Notes

Supplement 1. **eAppendix.** Interview Guide for Men Emergency Medicine Department Chairs

Supplement 2.
Data Sharing Statement
