# Supplementary material for: Physician Men Leaders in Emergency Medicine Bearing Witness to Gender-Based Discrimination
Source: JAMA Netw Open. 2023 Jan 5;6(1):e2249555. doi: 10.1001/jamanetworkopen.2022.49555 (PMC9857061; doi:10.1001/jamanetworkopen.2022.49555)
Supplement: Supplement 1. — eAppendix. Interview Guide for Men Emergency Medicine Department Chairs [file jamanetwopen-e2249555-s001.pdf]

Supplemental Online Content

Iyer MS, Wilson K, Draucker C, Hobgood C. Physician men leaders in emergency medicine bearing witness to gender-based discrimination. *JAMA Netw Open*. 2023;6(1):e2249555. doi:10.1001/jamanetworkopen.2022.49555

eAppendix. Interview Guide for Men Emergency Medicine Department Chairs

This supplemental material has been provided by the authors to give readers additional information about their work.

© 2023 Iyer MS et al. *JAMA Network Open*.

eAppendix. Interview Guide for Men Emergency Medicine Department Chairs

Interview Guide

1. Tell me about your role and responsibilities as chair.
    a. If the subject is not currently in the chair role modify the question to ask about chair leadership role.

2. How did you become a leader?
    a. If needed follow up with:
        i. Tell me about that process?
        ii. How did this evolve?
        iii. When did begin?

3. Tell me about your working environment?
    a. If the person has remained in the same institution ask "How did the environment change?"
    b. Make sure you identify:
        i. Colleagues? – "Who are the other leaders in your institution?"
        ii. The gender mix in the boardroom.
        iii. How do you interact with the other leaders in your institution?
    c. If the responses are closed, ask for examples.

4. How does it feel to be a [woman/ male] leader in your institution?

5. How have other [women/ men] reacted to you in your leadership role?
    a. Senior [women/ men]? Junior [women/ men]?
    b. How do you feel about that experience?

6. Tell me a story about a time when you felt like quitting?
    a. What prompted this?

7. Sexual Harassment has been defined by the EEOC as "including unwelcome sexual advances, requests for sexual favors, and other verbal or physical harassment of a sexual nature in the workplace or learning environment. Sexual harassment does not always have to be specifically about sexual behavior or directed at a specific person. For example, negative comments about women as a group may be a form of sexual harassment.
    a. What is your experience with sexual harassment in the workplace?
    b. How has your perspective on sexual harassment changed since you became a chair?
    c. Do you think you are more sensitive to these behaviors than those around you?

        Goal-- Explore-Sensitivity to issue, leadership management, and rationale for change

8. Mary Rowe of MIT defined micro-inequities as "small events which are often ephemeral and hard-to-prove, events which are covert, often unintentional, frequently unrecognized by the perpetrator, which occur wherever people are perceived to be 'different" in particular based on an unchangeable characteristic such as race or gender.
    a. Can you tell me about your experiences with this type of behavior?
    b. How did this make you feel, was that sensation immediate or delayed?
    c. Do you think you are more sensitive to these behaviors than those around you?

        Goal-- Explore-Sensitivity to issue, leadership management, and rationale for change

9. Microaggressions are hostile exchanges that send denigrating messages. They have been further defined as commonplace, daily, verbal, behavioral, or environmental indignities, whether intentional or unintentional, that communicate hostile, derogatory, or negative racial, gender, and sexual orientation and as religious slights and insults to a target person or group.
    a. Can you tell me about your experiences with this type of behavior?
    b. How did this make you feel?
    c. Do you feel you are more less sensitive to these behaviors than those around you? If so, how?

10. Tell me about your home life?
    a. Has leadership come with a personal cost or been a personal multiplier? How?

11. What are you going to do next?

© 2023 Iyer MS et al. *JAMA Network Open.*
