# Supplementary material for: Physician Men Leaders in Emergency Medicine Bearing Witness to Gender-Based Discrimination
Source: JAMA Netw Open. 2023 Jan 5;6(1):e2249555. doi: 10.1001/jamanetworkopen.2022.49555 (PMC9857061; doi:10.1001/jamanetworkopen.2022.49555)
Supplement: Supplement 2. — Data Sharing Statement [file jamanetwopen-e2249555-s002.pdf]

Data Sharing Statement

Iyer. Physician Men Leaders in Emergency Medicine Bearing Witness to Gender-Based Discrimination. *JAMA Netw Open*. Published January 05, 2023.
doi:10.1001/jamanetworkopen.2022.49555

Data
Data available: No

Additional Information
Explanation for why data not available: The data for this study will not be shared as our consent process ensured participants that all data would remain confidential. Data sharing of the original transcripts may allow identification of participants which would violate our pledge and the IRB approval.
